# QT Dispersion in Young, Ideal, and Old Aged Pregnancies

**Published:** 2014-01-01

**Authors:** Mohammad Hossein Nikoo, Shahdad Khosropanah, Soroush Alborzi, Amir Aslani

**Affiliations:** 1 Cardiovascular Research Center, Shiraz University of Medical Sciences, Shiraz, IR Iran

**Keywords:** Pregnancy, Maternal Age, High Risk Pregnancy

## Abstract

**Background:**

Obstetricians regard maternal age of 20 to 35 years as the optimal age for pregnancy. Adolescent pregnancy and pregnancy at the ages of 35 years and above are associated with higher risks. Pregnancy is pro-arrhythmic and rarely precipitates ventricular arrhythmias.

**Objectives:**

QT dispersion is an index of heterogeneity of ventricular repolarization and a predictor of propensity of ventricular arrhythmias. In this study, this index was used to find any relationship between maternal age and ventricular arrhythmia risk.

**Methods:**

This study was performed among a group of healthy pregnant ladies between 36 and 40 weeks of gestation. An ECG was taken from each patient. QT dispersions were calculated on a computer screen with high magnitude. The results were then divided into three groups based on the age of the participants. The first, second, and third groups included the women below 20, between 20 and 35, and over 35 years, respectively. The three groups were compared using Kruskal-Wallis test.

**Results:**

The mean QTd was 61.77 ms (± 16.61) in the first group, 64.15 ms (± 18.65) in the second group, and 55.95 ms (± 23.04) in the third group. Although QTd was prolonged in all, no significant difference was observed among the three groups regarding QTd.

**Conclusions:**

Our results showed QT prolongation in pregnancy, but showed that maternal age did not affect the heterogeneity of ventricular repolarization and propensity of ventricular arrhythmias in pregnancy.

## 1. Background

Pregnant women are one of the most vulnerable target groups in all healthcare systems and their wellbeing is regarded with utmost importance by global, national, and regional health authorities around the world. Both World Health Organization (WHO) and Iran's ministry of health have also put a great emphasis on improving the standards of care for pregnant women and reducing the risks associated with pregnancy.

One of the factors associated with higher mortality and morbidity in both women and their children is pregnancy in extremes of fertile ages. It is a longstanding tradition backed by numerous studies in obstetrics to define the optimal maternal age for pregnancy as between 20 and 35 years and regard pregnancies with the maternal age of lower than 20 or higher than 35 to be associated with a high risk (1). For instance, pregnancy in adolescence is associated with a higher incidence of anemia, restricted infant growth, and preterm labor and a higher infant mortality rate (1, 2). On the other hand, pregnant women who are above 35 years old are more likely to experience preterm labor, low infant birth weight, placental abruption, placenta previa, hypertension, and diabetes (1). These women also have a higher maternal mortality rate (3).

Pregnancy has a huge effect on the cardiovascular system. It increases blood volume, heart rate, venous pressure in lower extremities, and cardiac output. In addition, it decreases peripheral resistance and pulmonary vascular resistance and may also lower the blood pressure (4). It also affects the conduction system of the heart, making patients more susceptible to arrhythmias. The most common arrhythmias in pregnancy are atrial ones; however, ventricular tachyarrhythmia does rarely happen during pregnancy and could be life-threatening (4, 5).

There has been a lot of research revolving around the changes in the cardiac conduction system during pregnancy and we will discuss some of them later. Yet, the literature is silent about the possible effects of the age of pregnant women on these changes. It seems worthwhile to find out if there is any difference between the women in the optimal age for pregnancy and those in the two above-mentioned extremes regarding conduction disturbances.

QT dispersion on surface electrocardiogram is a long-established indicator of heterogeneities of ventricular repolarization and a reasonably reliable predictor of vulnerability to ventricular arrhythmias (6). It is a low-cost and noninvasive index and is therefore used in various studies as an indirect tool to show the susceptibility of the patients to develop ventricular arrhythmias. Some of these studies will be discussed later.

Given the arrhythmogenic effects of pregnancy, it is not surprising that studies have found QT dispersion to be higher among the pregnant women compared tothe non-pregnant ones (7). However, the effects of age on this change are still unknown. Thus, the present study aims to determine whether pregnancy in the extremes of age has any effects on this important electrocardiographic index.

## 2. Patients and Methods

This study was performed on a group of pregnant ladies who had referred to two private obstetric clinics in Shiraz. We thoroughly explained the study and its goal to these women and then asked if they would like to collaborate in this study. Those who were willing to participate in our study were provided with more detailed information and all their questions were answered. They were then asked to sign the written consents and were entered into the study.

The volunteers were asked about any history of heart disease or consumption of medication. Those who had a negative history of cardiac illness and medication consumption were asked to come back when their pregnancy age, as determined by their last menstrual period, was between 36 and 40 weeks. The ladies who were not able to determine their last menstrual periods or were in doubt were excluded from the study.

Some of the participants did not refer to us at the appropriate time, but many of them did. The individuals who did refer on time were each assigned a random three-digit number and were asked about their age. The assigned number and the corresponding age were recorded in a notebook. Afterwards, a standard 12-lead electrocardiogram was taken from each patient and the assigned number was marked on the ECG. No name or any other personal information was mentioned on the ECG.

These electrocardiograms were collected and carefully scrutinized. The ECGs with too many artifacts or with signs of arrhythmia, atrioventricular block, bundle branch block, ischemia, or infarction were excluded from the study. The remaining ECGs were used for our investigation and QT dispersion was measured in every ECG.

In order to calculate QT dispersion, we first scanned the ECGs and then viewed them on a computer screen with high magnitude. After that, we used MathLab software to measure the distance between the start of the Q wave and the end of the T wave in two consecutive beats in each standard lead. The mean of these two distances was regarded as the QT interval in that lead. The end of T wave was determined as the point where T wave returns to the level of TP line. When U wave was present, the QT interval was measured to the nadir of the curve between T and U waves. The leads with blurred-ended or flat T waves were neglected. Each ECG would be deemed acceptable for the purpose of our study if QT interval was measurable in at least 9 leads. The mean QT interval in each lead was then corrected using Bazett's formula as follows: QTc = QT / √RR, with QTc being the corrected QT. Afterwards, the QTc dispersion in each ECG was computed by subtracting the shortest QTc from the longest one (8, 9).

Using this method, we calculated QTc dispersion in all the acceptable ECGs. We then divided the results into three groups based on the age of our participants. The first group consisted of the women under the age of 20. The second group included all the women between 20 and 35 years old and the last group was made up of the women who were 35 years of age or older. Finally, Kruskal-Wallis test was employed to analyze the data.

## 3. Results

Overall, 234 participants had acceptable ECGs in which QTc dispersions were calculated. Among these participants, 67 (28.6%), 98 (41.9%), and 69 ones (29.5%) were allocated into the first, second, and third age groups, respectively.

We considered the maximal measured amount of corrected QT intervals in the standard leads in each ECG as QTc in that ECG. The mean of QTcs was 424.04 + 17.43, 424.06 + 21.43, and 425.08 + 15.45 ms in the first, second, and third groups, respectively. The results showed no statistically significant difference among the three groups regarding the QTcs.

In the first group, the minimum and maximum QTc dispersion stood at 38 and 93 ms, respectively. Besides, the mean QTc dispersion was 61.77 + 16.61 ms.

In the second group, we observed a minimum QTc dispersion of 33 and a maximum of 123 ms. In addition, the mean QTc dispersion was 64.15 + 18.65 ms.

Finally, in the third group, the minimum QTc dispersion was 25 and the maximum was 104 ms. Also, the mean QTc dispersion was 55.95 + 23.04 ms.

The results of Kruskal Wallis test revealed no statistically significant difference among the three study groups regarding the QTc dispersions. It should be noted that the confidence interval of this test was 99%.

## 4. Discussion

As mentioned earlier, the risk and prevalence of many types of arrhythmia are increased during pregnancy (4, 5). This is usually attributed to the hemodynamic, autonomic, and hormonal alterations in pregnancy (10). Moreover, Lechmanova et al. have shown that QT dispersion, which is an index of heterogeneity of ventricular repolarization and a powerful predictor of propensity of ventricular arrhythmias, increased during pregnancy (7).

We also discussed the longstanding tradition in obstetrics to regard the maternal age of 20 to 35 years as the optimal age for pregnancy and other maternal age groups as high risk (1-3). However, the present study revealed no significant difference between the women in the optimal maternal age and those in the two extremes of reproductive ages regarding the QT dispersion.

This finding suggests that in healthy pregnant women, age and its related physiological differences do not affect the dispersion of ventricular repolarization and susceptibility to develop ventricular arrhythmias. In other words, it can be claimed that age cannot be considered as a risk factor in predicting the likelihood of ventricular arrhythmias in healthy pregnant women.

This may be due to the nature of reproductive age in women. Generally, most pregnant women are teenagers or young or middle-aged ladies. They are neither too old nor too young. They are therefore at a low risk for problems, such as congenital heart disease, which are usually detected in children. They are also at a low risk for the problems which are more commonly associated withold age, such as congestive heart failure and coronary artery disease.

One other possible explanation may be that the physiological changes that accompany pregnancy far outweigh any differences produced by maternal age. As mentioned above, pregnancy is associated with major autonomic and hemodynamic changes. The overall effects of these changes are stretching of myocardium and increased sympathetic tone, both of which may contribute to generation and propagation of arrhythmias (10). We also said that many studies have revealed the existence of various steroidal sex hormone receptors in the heart. This suggests that the significant hormonal changes that occur during pregnancy may have a huge influence on the heart and contribute to the proarrhythmic effect of pregnancy (11, 12). The sum of all these arrhythmogenic mechanisms may overshadow any possible effects of maternal age on the conduction system.

The findings of the current study emphasized QT prolongation during pregnancy although no significant difference was found among the three groups regarding QT intervals. This supports our hypothesis that the effects of maternal age on ventricular conduction system are negligible. However, further investigations are required before we can cast the final judgment on this issue.
